# Protease Inhibitors Purified from the Canola Meal Extracts of Two Genetically Diverse Genotypes Exhibit Antidiabetic and Antihypertension Properties

**DOI:** 10.3390/molecules26072078

**Published:** 2021-04-04

**Authors:** Saira Hussain, Ata ur Rehman, David J. Luckett, Syed Muhammad Saqlan Naqvi, Christopher L. Blanchard

**Affiliations:** 1 Graham Centre (an Alliance between Charles Sturt University and the NSW Department of Primary Industries), Boorooma Street, Wagga Wagga, NSW 2678, Australia; djluckett@gmail.com (D.J.L.); saqlan1472@gmail.com (S.M.S.N.); CBlanchard@csu.edu.au (C.L.B.); 2 ARC Industrial Transformation Training Centre for Functional Grains, School of Biomedical Sciences, Charles Sturt University, Boorooma Street, Wagga Wagga, NSW 2678, Australia

**Keywords:** canola meal, protease inhibitor, dipeptidyl peptidase-IV, angiotensin converting enzyme

## Abstract

Valorization of vegetable oil waste residues is gaining importance due to their high protein and polyphenol contents. Protease inhibitors (PIs), proteins from these abundantly available waste residues, have recently gained importance in treating chronic diseases. This research aimed to use canola meal of genetically diverse *Brassica napus* genotypes, BLN-3347 and Rivette, to identify PIs with diverse functionalities in therapeutic and pharmacological applications. The canola meal PI purification steps involved: native PAGE and trypsin inhibition activity, followed by ammonium sulfate fractionation, anion exchange, gel filtration, and reverse-phase chromatography. The purified PI preparations were characterized using SDS-PAGE, isoelectric focusing (IEF), and N terminal sequencing. SDS-PAGE analysis of PI preparations under native reducing and nonreducing conditions revealed three polymorphic PIs in each genotype. The corresponding IEF of the genotype BLN-3347, exhibited three acidic isoforms with isoelectric points (p*I*) of 4.6, 4.0, and 3.9, while Rivette possessed three isoforms, exhibiting two basic forms of p*I* 8.65 and 9.9, and one acidic of p*I* 6.55. Purified PI preparations from both the genotypes displayed dipeptidyl peptidase-IV (DPP-IV) and angiotensin-converting enzyme (ACE) inhibition activities; the BLN-3347 PI preparation exhibited a strong inhibitory effect with lower IC_50_ values (DPP-IV 37.42 µg/mL; ACE 129 µg/mL) than that from Rivette (DPP-IV 67.97 µg/mL; ACE 376.2 µg/mL). In addition to potential human therapy, these highly polymorphic PIs, which can inhibit damaging serine proteases secreted by canola plant pathogens, have the potential to be used by canola plant breeders to seek qualitative trait locus (QTLs) linked to genes conferring resistance to canola diseases.

## 1. Introduction

Plants, being sessile and host to a full spectrum of biotic threats, have developed an elaborate and elegant chemical defense arsenal of phyoproteins, with protease inhibitors (PIs) being an important component [1,2]. PIs are primarily present at high concentrations in plant storage organs such as seeds [3]. They are known for regulating endogenous plant proteases and, therefore, act as biopesticides and antifungal agents [4,5]. PIs also have potential applications in the treatment of many human conditions, including cancer [2,6], blood clotting disorders [7,8], AIDS [9], neurodegenerative disorders [10], hypertension [11], and obesity [12,13]. 

There have been several studies on *Brassica napus* proteins and peptides [14] and other reports on PIs in the wider Brassicaceae family [15]; however, the diversity of *B. napus* (canola) PIs and polyphenols and their activities and possible applications have not been comprehensively explored using canola meal. 

Canola meal is an abundantly available waste product of oil extraction from *Brassica napus* seed and is considered a low-value product. This meal may have high value in the biotechnology, bioceutical, and biomedical fields if potential health-beneficial bioactive compounds can be identified with the ability to prevent or alleviate significant modern-day illnesses, including obesity, diabetes mellitus, and hypertension. 

Diabetes mellitus (DM) is a common debilitating human illness worldwide [16], with an increasing rate of occurrence [17]. Type-II diabetes typically involves a gut hormone known as glucagon-like peptide-1 (GLP-1). It regulates diabetes via its dual role for the stimulation of insulin release from beta-cells and reduction of glucagon secretion [18]. Dipeptidyl peptidase-IV (DPP-IV, EC 3.4.14.5) is a serine protease [19], which inactivates GLP-1 [20] within 1–2 min of entering blood circulation [21]. Research on animals with a genetic loss of DPP-IV showed better tolerance to glucose and high rates of insulin secretion [22]. Hence, natural inhibitors of this protease may enhance the amount of endogenous active GLP-1 and insulin secretion [23]. 

Angiotensin-converting enzyme (ACE) is a carboxydipeptidase (EC. 3.4.1.5.1), having a major role in controlling blood pressure and salt-water balance by converting angiotensin-I into -II. It is also implicated in the deactivation of bradykinin, a hypotensive peptide [24]. Elevated blood pressure is, therefore, often treated with ACE-inhibiting drugs [25]. PIs from natural sources may provide an alternative treatment for ACE-inhibition regulation of blood pressure. Previous studies have identified peptides from whey and canola meal hydrolysates showing antihypertensive and ACE-inhibition activities [26,27]. 

Our recent studies on canola meal extracts have established antiobesity properties of canola meal biophenols [16]. However, the main objective of this study was to identify and characterize individual PIs from canola meal, and to determine their possible use for therapeutic purposes for inhibition of DPP-IV and/or ACE.

## 2. Results

### 2.1. Extraction and Purification

**Figure 1:**
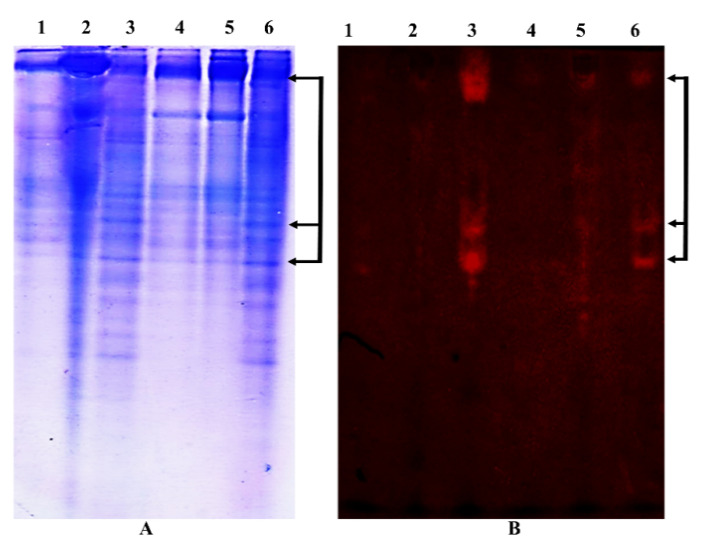
Native gel electrophoresis: (**A**) native-PAGE of canola meal crude extracts after staining with Coomassie brilliant blue and (**B**) corresponding native-PAGE gel showing trypsin inhibitor activity (enzymatic staining). Protein bands with PI activity are indicated by arrows. Lanes: 1, 2, and 3 show 25%, 50%, and 80% saturated ammonium sulphate precipitated (AS) fractions from BLN-3347, respectively; while lanes: 4, 5, and 6 show 25%, 50%, and 80% (w/v) AS fraction from Rivette, respectively.

The presence of trypsin inhibitory activity in the canola meal crude extracts at 50–80% ammonium sulfate saturation from both canola genotypes, Rivette and BLN-3347, was established spectrophotometrically and via trypsin staining of the gels after native PAGE of the extracts. No significant α-chymotrypsin inhibitory activity was observed in the crude extracts, and thus the α-chymotrypsin assay was not carried out in the subsequent purification steps. Supernatant fractionated at 50–80% ammonium sulfate saturation showed the most significant trypsin inhibitor activity and was used for further purification of PIs (Figure 1).

**Figure 2:**
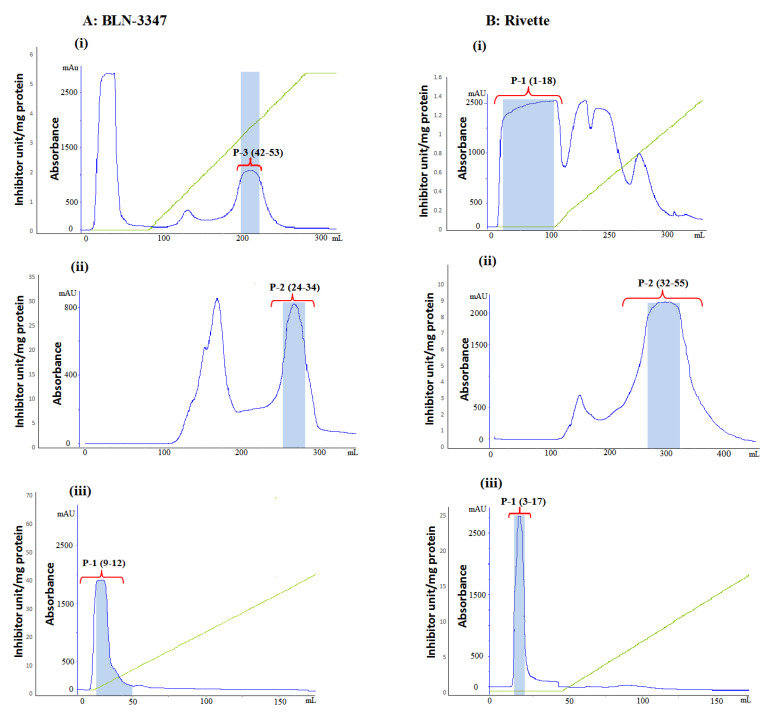
FPLC elution profiles of PIs from canola meal extracts of (**A**) BLN-3447 and (**B**) Rivette. Numbers in the bracket at the top of each peak represent fraction numbers obtained in respective peaks. (**A i**) 80% ammonium sulfate fraction separated by ion-exchange chromatography, showing active protease inhibitor (PI) peak, P-3. (**A ii**) Concentrated ion-exchange peak P-3 separated on gel filtration column showing active PI peak, P-2. (**A iii**) Concentrated active peak P-2 from gel filtration column separated on ion-exchange column, showing the active peak, P1. (**B i**) 80% ammonium sulfate fraction separated by ion-exchange chromatography, showing active PI peak, P-1. (**B ii**) Concentrated ion-exchange peak P-1 separated on gel filtration column showing an active PI peak, P-2. (**B iii**) Concentrated active peak P-2 from gel filtration column separated on the ion-exchange column, showing the active peak, P-1.

Figure 2 shows the results of FPLC analysis of canola meal (CM) extracts of genotypes BLN- 3347 and Rivette. Among the different protein peaks observed in the primary anion exchange chromatography (AEC), only P-3 from fractions 42 to 53 exhibited PI activity in BLN-3347 (Figure 2 Ai). Consequently, the fractions corresponding to this peak were pooled. In contrast, the PI activity in the Rivette sample was only observed in the unbound eluted fractions 1 to 18, shown under the peak P-1 (Figure 2 Bi). These fractions were also pooled and stored for further purification.

The pooled fractions from the active peaks from both genotypes were applied to a gel filtration chromatography column. The BLN-3347 proteins separated into two similarly sized peaks with only the second peak, P-2, showing PI activity in fractions 24 to 37 (Figure 2 Aii). The Rivette mixture also eluted into two peaks with a broad active peak, P-2, consisting of fractions 32 to 55 (Figure 2 Bii). The active pooled fractions from BLN-3347 and Rivette samples from gel filtration were rechromatographed separately onto the AEC column. The BLN-3347 fraction revealed a PI active peak, P-1, corresponding to fractions number 9 to 12 (Figure 2 Aiii). Rivette also showed one very distinct peak, P-1, corresponding to fractions 3 to 17 (Figure 2 Biii). While Figure 2, shows protease inhibitor activity assays of all protein fractions, the data in Table 1 complement these results by tracking the purification steps from crude CM extracts through to purified fractions.

The AEC fractions separated by gel filtration showed two major protein peaks. However, only one peak of the two exhibited trypsin inhibition activity in each genotype (Figure 2 Aii and Bii). Peaks that were pooled and reapplied to AEC revealed a doubling of specific activity for BLN-3347 and a tripling for Rivette (Figure 2 Aiii and Biii). 

**Figure 3:**
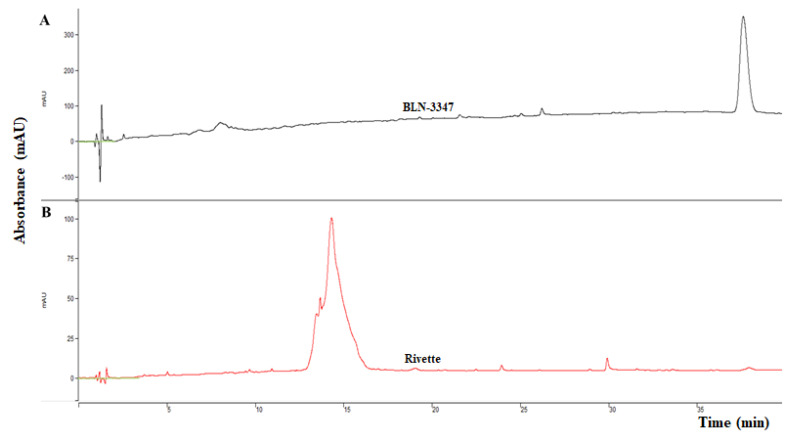
RP-HPLC (214 nm) elution profiles of canola protease inhibitor samples from BLN-3347 (**A**) and Rivette (**B**). Absorbance (mAU) was measured at 214 nm by Y-axis. Retention time in minutes (min) was measured along with fraction collection by X-axis.

Finally, the pooled active fractions from the second AEC were subjected to reverse phase chromatography (RPC), where BLN-3347 eluted only one peak (Figure 3A). Rivette showed two small overlapping peaks within one large peak, implying the presence of more than one molecule (Figure 3B). Both BLN-3347 and Rivette fractions did not show any significant change in the specific activity.

The RP-HPLC revealed a single major PI peak for BLN-3447, whereas Rivette exhibited a major peak along two small shoulder peaks. Both PI preparations from the pooled fractions under these peaks were used to determine DPP-IV and ACE inhibition activities.

The *N*-terminal sequences reported in this study are tentative/probable *N*-terminal sequences deduced from the raw data. However, to deduce the peptide sequence of individual PIs and to ascertain their role in DPP-1V and ACE inhibitions, the two preparations should be subjected to iso-electro focusing followed by MALDI-MS sequence analysis. Since the separation of individual PIs requires massive amounts of canola meal as starting material, this regard is considered a separate research endeavour.

### 2.2. DPP-IV Inhibition and ACE Inhibition 

**Figure 4:**
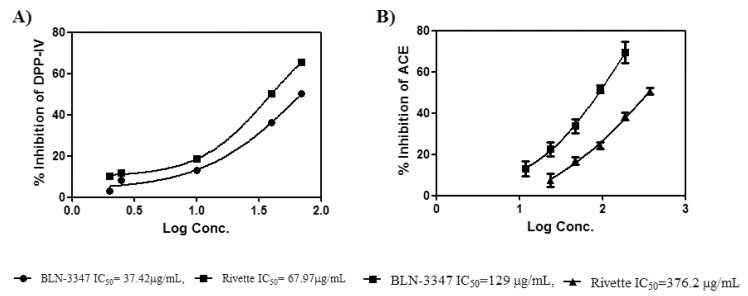
(**A**) DPP-IV (dipeptidyl peptidase-IV) inhibition activity of protease inhibitors (PIs) extracted from defatted meal from two different canola genotypes. (**B**) ACE (angiotensin converting enzyme) inhibition activity of the protease inhibitors obtained from the same canola meals. The calculated IC_50_ values were read from the fitted nonlinear regression lines and are given in the key below the graph frames. Error bars indicate ± the standard error at each point.

IC_50_ values for DPP-IV and ACE inhibition of the PI preparations are shown in Figure 4. Samples from both the genotypes showed inhibitory activity but BLN-3347 appeared superior to Rivette. Similarly, the IC_50_ values of ACE inhibition were also determined by plotting the log of concentration against percentage inhibition (Figure 4B). 

The ACE inhibition was much less pronounced compared to DPP-IV inhibition, but the relative effectiveness of both the genotypes was the same, with BLN-3347 showing almost three times more inhibition than Rivette (Figure 4).

### 2.3. Molecular Characterization of Purified PIs

**Figure 5:**
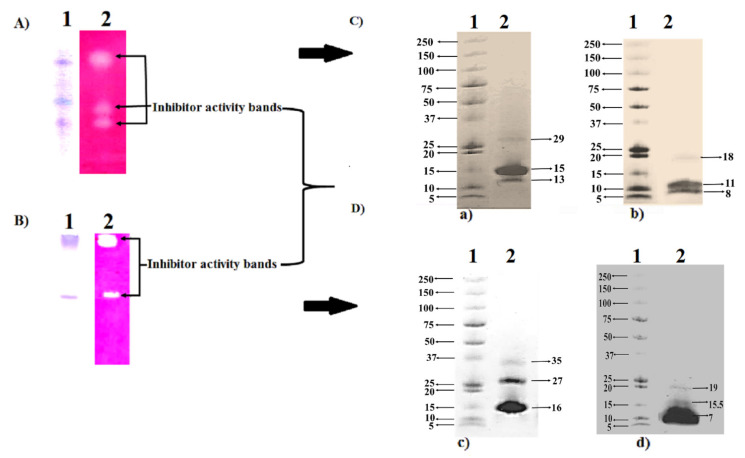
PAGE analysis of RP-HPLC fractions (**A**) (BLN-3347) and (**B**) (Rivette). (**A**,**B**) PI activity-stained native-PAGE gel and corresponding (**C**,**D**) Coomassie brilliant blue-stained SDS-PAGE gel under (**a**,**c**) nonreducing and (**b**,**d**) reducing conditions. Lanes: 1 = Precision Plus Protein™ Dual Xtra Standard marker; 2 = canola PIs.

Both RP-HPLC PI active peaks from BLN-3347 and Rivette were resolved on native-PAGE gels, exhibiting three trypsin inhibitory bands per genotype (Figure 5A,B). Similarly, the SDS-PAGE of the fractions generated three bands of different molecular sizes under nonreducing conditions, which were further reduced in size under reducing conditions (Figure 5C,D). The fact that Rivette PIs showed no contaminating bands on SDS-PAGE gels, despite their anionic nature, could well be attributed to the gel filtration step.

**Figure 6:**
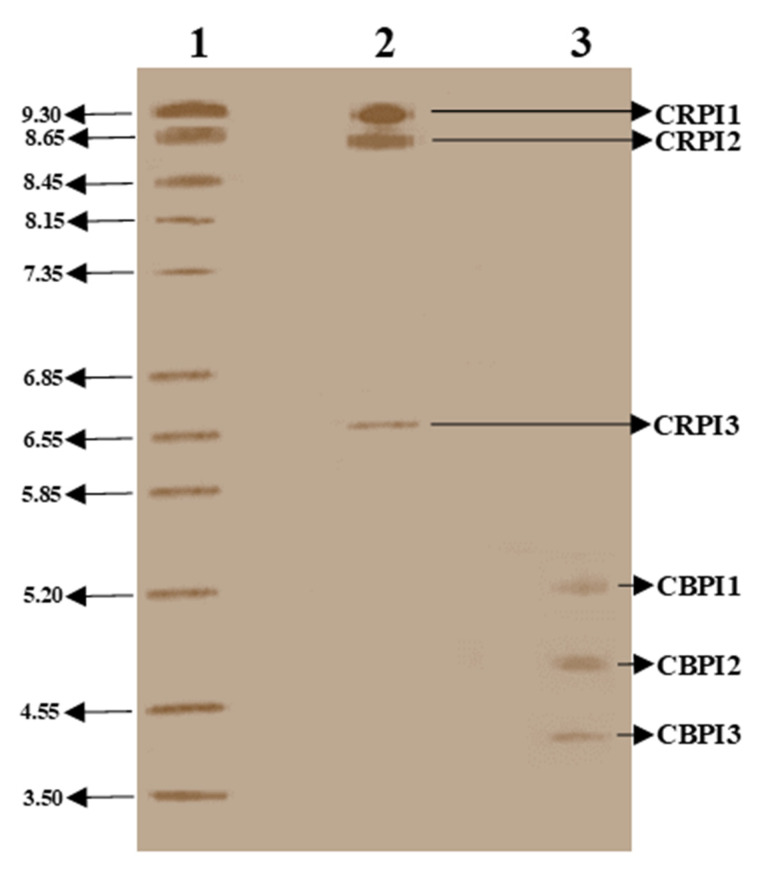
Isoelectric focusing gels (IEF gels 3–9 [pH range 3–9, Pharmacia]) were used for the purified protease inhibitors. Lanes 1 = markers with different isoelectric points (p*I*) ranging from 3 to 10, 2 = Rivette PIs, and 3 = BLN-3347 PIs.

Furthermore, isoelectric points (p*I*) were determined by running IEF gels. BLN-3477 revealed isoforms with p*I* values of 4.0, 4.60, and 4.90, labeled as CBPI, CBP2, and CBP3, respectively, while the Rivette showed isoforms with p*I* values of 9.30, 8.65, and 6.55, labeled as CRPI11, CRPI12, and CRPI13, respectively (Figure 6).

The *N*-terminal amino acid sequences of the three protein subunits of BLN-3347 and Rivette were determined and aligned to homologous sequences contained in public databases using BLAST software. Additionally, their experimentally calculated isoelectric points and molecular weights were also compared to those of aligned sequences in the public database (Table 2). 

Table 2 reveals bioinformatics analysis, showing the first protease inhibitor in BLN-3347, an 8 kDa protein subunit sequence with a calculated value of 6.4 kDa. This was recognized as homologous to epithiospecific protein [*Brassica napus*] with accession number XP_013643640.1 and a calculated p*I* of 4.49, while the observed p*I* was equal to 4.00. The second 11 kDa sequence was shown to have a calculated value of 11.5 kDa, with the matching sequence, accession number of XP_013710061. It consisted of about 100 amino acids and was identified as a putative *Lactoylglutathione lyase* [*Brassica napus*]. This protease inhibitor had a calculated p*I* of 5.37, while the observed p*I* was 6.13. The third 18 kDa sequence showed no complete homology with any database sequences and thus may be treated as a new molecule. 

The Rivette 7 kDa molecule had a calculated value of 5.11 kDa, and was identified as a defensin-like protein 4 [*Brassica napus*] with accession number XP_013643640.1. This protease inhibitor had a calculated p*I* of 8.92, while the observed p*I* was 8.65. The Rivette second 15.5 kDa sequence had a calculated value of 9.813 kDa, with accession number P80208.1, known as 2SS3_BRANA Napin-3 OS = *Brassica napus* PE = 1 SV = 1. This protease inhibitor had a calculated p*I* of 9.15, while the observed p*I* was 9.30.

## 3. Discussion

The presence of a few PIs in the seed of rapeseed has been known for some time [28], however, there has been no study for PIs in canola meal. While the presence of small DPP-IV and ACE inhibition peptides has been recently reported in other species [29], there has been no report on canola meal extracts. Moreover, neither the individual PIs nor their polymorphisms have been reported in detail. More recently, different oilseed proteins, including from rapeseed, have demonstrated the presence of ACE- and DPP-IV-inhibitory peptides [30,31]. In this study, canola meal from two genetically diverse genotypes was used to discover six protease inhibitors, which were evaluated for their potential antidiabetic and antihypertension potential. Expedition to find molecular targets and mechanisms of action of plant-based bioactive peptides is needed to use these bioactive peptides as a potential drug candidate. Separate unpublished studies from our lab have also shown purified phenolic compounds from canola meal exhibiting antioxidant, antidiabetic, and antiadipogenic properties, concomitantly stripping major antinutritional compounds such glucosinolates, phenolics, and phytates, a process which could make the processed meal a potentially better source of protein-rich food for humans and animals. 

The anion exchange chromatography of BLN-3347 CM crude extract at 50–80% ammonium sulfate saturation eliminated most of the carbohydrates and other residual contaminants. On the other hand, the cation exchange chromatography of the Rivette active PI-1 fraction resulted in consistent protein precipitation, creating cracks in the packed column, perhaps due to the nonspecific interaction of the column resin proteins. Consequently, the Rivette CM ammonium sulfate fraction was resolved on the anion exchange column. This step may have carried over both proteinaceous and nonproteinaceous contaminants, even after gel filtration and the second AEC step. However, the last RP-HPLC and SDS-PAGE of the RP-HPLC fraction revealed no contaminating peaks and bands, respectively. 

The SDS-PAGE analysis of the RP-HPLC fractions revealed three individual protein bands in both BLN-3347 and Rivette, with an apparent molecular mass of 29, 15, 13 kDa and 37, 27,16 kDa, respectively. However, in the presence of reducing reagents, these bands were reduced to 18, 11, 8 kDa and 19, 15.5, 7 kDa, respectively, suggesting that the purified fraction probably comprised two polypeptide chains, linked by one or more disulfide bridges. The other smaller chain of polypeptides for each of these was presumed to have run off the gel or was too faint to be detected as it was not observed from SDS-PAGE analysis. Since none of these identified polypeptides were common between the two genotypes, the range of available PIs in the whole canola gene pool may be large. Despite having different molecular weights and p*I*s, the elution of these proteins in the same fraction in all the four chromatographic steps is quite intriguing and may be due to some type of association between them, hitherto unexplored. More than one PI polypeptide has also been reported in protein purification from wattle seeds [32]. 

The molecular mechanism of action of a bioactive compound can be termed as the molecular interactions between its therapeutic treatment and the biological target that yield the physiological response. In this regard, IC_50_ is an informative measure of the effectiveness of any bioactive compound against the biological target. This study explored canola PIs for DPP-IV inhibitory activity and observed a dose-dependent inhibition of DPP-IV by both PI preparations from BLN-3347 and Rivette. Although both PIs were adequate, the kinetics showed that the inhibitory concentration (IC_50_) of PI preparation from canola genotype BLN-3347 had values 1.8 times less than Rivette for DPP-IV inhibition activity (Figure 4A). This study also demonstrated the presence of PIs with ACE-inhibitory activity in canola seeds, suggesting a possible application of canola extracts for research into the control of blood pressure through the inhibition of ACE. The two genotypes, BLN-3347 and Rivette, differed in their potency, suggesting the choice of genotype would be an essential factor if canola meal or its extracts were to be used as a food ingredient, a therapeutic agent, or as a source of PI extraction. 

The ACE inhibition IC_50_ values of the purified PI preparations of two canola genotypes, purified in this study, were compared with the IC_50_ values of compounds from different species. These compounds included in vitro digested dry fruit bodies of *Pleurotus ostreatus* (IC_50_ 510 μg/mL) [31], yeast hydrolyzates (26.13 μg/mL [32], and chickpea accession BDN-9-3 (IC_50_ 22430 μg/mL [33]. Considering these values, canola meal PIs in our studies showed significant potential for ACE inhibition. 

Similarly, DPP-IV inhibition IC_50_ values of PI preparation from genotype BLN-3347 (129 ug/mL) and Rivette (376.2 ug/mL) indicated this as a potent candidates for the treatment against diabetes mellitus when compared with the IC_50_ values of compounds from several different species, including soy protein peptides (2730 μg/mL) [34], two protein hydrolysates from microalgae (2280 and 2680 μg/mL) [35], α-lactalbumin hydrolysate (36 μg/mL) [36]**,** and Napin-derived hydrolysate of rapeseed 680 μg/mL [36,37].

The family of Kunitz-type protease inhibitors is well known, and they are common in higher quantities in plant seeds [33] and are generally more than 8 kDa in size. Since all the sequences identified here were more than 8 kDa, except one, it seems likely that most of them belong to the Kunitz-type trypsin inhibitor family. PIs from *Brassicaceae* have also been known for their function in plants under environmental stresses, such as salinity and drought [34]. 

Canola protein extraction has a low recovery rate due to different isoelectric points and molecular weights, as reported in [35]. Therefore, relatively large amounts of the meal are required to generate sufficient protein for analysis (Table 1) of protease inhibitors. Work is in progress to obtain enough protein from Rivette and BLN-3447 to separate individual isoforms using preparative isoelectric focusing, and to ascertain their specific role in the inhibition of DPP--IV and carboxydipeptidase enzymes.

## 4. Materials and Methods

Canola seeds of the two genotypes, BLN-3347 and Rivette, were used in this study. Rivette is an open-pollinated, Australian commercial cultivar (now outclassed), and BLN-3347 is an unreleased breeding line from the New South Wales Department of Primary Industries’ (NSW DPI) Australia breeding program. These genotypes were chosen as they were the parents of a doubled-haploid population, which could subsequently be used to study the inheritance of any valuable PIs identified in the study.

Chemicals, including ammonium sulphate, calcium chloride (CaCl_2_), tris hydrochloride (Tris-HCl), glycerol, bromophenol, *N*-acetyl-dl-phenylalanine β-naphthyl ester (APNE), tetrazoitized (zinc chloride complex) o-dianisidine (Fast blue B salt), bovine trypsin, soybean trypsin inhibitor, *p*-toluenesulfonyl-L-arginine-methyl ester (TAME), sodium dodecyle sulphate (SDS), ammonium persulfate (APS), *N*,*N*,*N*′,*N*′-tetramethylethylenediamine (TEMED), *β*-mercaptoethanol (ME), *N*,*N*-dimethylformamide, Trifluroacetic acid (TFA), and colour marker, were purchased from Sigma Aldrich, Castle Hill, NSW, Australia. Polyacrylamide precast gels (PhastGel gradient 8-25, Homogeneous 20 and IEF), PhastGel buffer strips, PhastGel Blue R tablets, and PlusOne silver staining kit were purchased from GE Healthcare, Uppsala, Sweden. Ultrapure water (UPW) with 0.22 μm filter was supplied by Thermo Fisher Scientific, Victoria, Australia. 

Canola meal (CM) was prepared by grinding 100 g of the whole, cleaned seed in a mechanical grinder (Foss Knifetec^TM^ 1095, Slangerupgade, Denmark) and the oil was then removed using 80 mL of absolute *n*-hexane for 16 h in a soxhlet rotovap machine (Soxtec™ 2050, Tector™ Technology, Slangerupgade, Denmark) at the Australian Oilseed Laboratory, NSW DPI, Wagga Wagga, Australia. The defatted meal was dried under a ventilated fume hood overnight at room temperature.

CM (180 mg/mL) from each genotype was dissolved in extraction buffer (0.023 M CaCl_2_, 0.092 M Tris-HCl at pH 8.1), centrifuged at 112× *g* for 3 min (Hermle; Gosheim, Germany), filtered to remove debris, and further cleaned by passing through a Bio-Spin® chromatography column (Bio-rad column). Total protein was estimated by using a Pierce™ BCA Protein Assay Kit (Thermo Scientific, Brisbane, Australia). The presence of PIs was determined by inhibition activity assays. 

### 4.1. Isolation and Purification of Canola Meal Protease Inhibitors

CM samples (400 g) of each genotype were separately mixed in a 1:10 ratio with ultrapure water (UPW) at 4 °C for 5 h with constant stirring, followed by centrifugation at 8200× *g* for 30 min. The supernatant was collected and sequentially precipitated under constant stirring in 25%, 50%, and 80% ammonium sulphate (NH_4_SO_4_) saturation. Each precipitate was dissolved in a minimal amount of Ultrapure water (UPW) and dialyzed for 24 h at room temperature before concentrating with Amicon Ultra-15 centrifuge filters (Merck Millipore, Australia). The concentrated fraction was loaded on a HiPrep 16/10 Q-Sepharose Fast Flow anion exchange column (GE Healthcare Bio-Sciences, Uppsala, Sweden), pre-equilibrated with Tris-HCl (50 mM) buffer at 20 °C, pH 7.0, at a flow rate of 1 mL/min. Fractions of 5 mL were eluted with a linear gradient of NaCl (0.0–0.5 M) in the same buffer. The active fractions under a protein peak were pooled and concentrated using Amicon Ultra-15 Centrifuge tubes (10 kDa cut-off). The concentrated retentate was then loaded onto a Hi-Load 26/60 Superdex 200 size exclusion column (GE Healthcare Bio-Sciences, Uppsala, Sweden), pre-equilibrated with 50 mM Tris-HCl buffer (pH 7.0) at a flow rate of 1 mL/min at 20 °C. All the active fractions under each protein peak were concentrated again using the Amicon Ultra-15 Centrifuge filter. The concentrated fractions were then rechromatographed using the same anion exchange column with the same conditions, and the pooled active fraction was finally subjected to reverse phase chromatography on HPLC Luna analytical C18 RP column (i.d. = 3 mm, 150 mm long, with a 10 μm particle size) pre-equilibrated with 0.1% (*v*/*v*) trifluoroacetic acid (TFA) in UPW. Protein separation was achieved using a linear acetonitrile gradient of 1−100% in 0.1% (*v*/*v*) TFA for 40 min at a flow rate of 1 mL/min. The HPLC was equipped with a 214 nm UV detector. 

#### 4.1.1. Protease Inhibitor (PI) Activity Assay

Assays for bovine trypsin and α-chymotrypsin inhibitors were carried out as previously described [36,37], by estimating the remaining esterolytic activity of trypsin and chymotrypsin using *p*-toluenesulphonyl-L-arginine methyl ester (TAME) and *N*-benzoyl-*L*-tyrosine ethyl ester (BTEE), respectively. Soybean trypsin inhibitor (Sigma) and wattle seed extract [32] were used as a control. One trypsin unit (TU) or α-chymotrypsin unit (CU) is defined as 1 μmol of substrate hydrolyzed per minute of reaction. One inhibition unit is defined as a unit of enzyme inhibited. Trypsin and α-chymotrypsin inhibitor activity assays were carried out in every step of purification to identify the specific activity of inhibitors. Specific activity is defined as trypsin inhibition units (TIU) per milligram of protein.

Protease inhibitor activity of the different fractions obtained during the PI purification steps was measured spectrophotometrically only using trypsin as a substrate and by visualizing trypsin-inhibiting PI bands, which had been resolved on native-PAGE gels.

#### 4.1.2. In-Gel Trypsin Inhibitor Activity

A duplicate set of CM crude extract and fractions from all stages of purification was treated with Laemmli’s buffer without SDS and β-mercaptoethanol. All samples were incubated at room temperature before loading onto the 2 × 12% native-PAGE gels using Biorad Protean II XL Electrophoresis Cell (Bio-Rad Laboratories Pty Ltd, Gladesville, Australia). Native marker high molecular weight (HMW) 66 to 669 KDa (GE Healthcare, Bioscience, Uppsala, Sweden) was also loaded along with the samples and soybean inhibitor (positive control). Precision Plus Protein™ Dual Xtra Standard marker 2–250 KDa (Biorad, Australia) [38] and Color Marker low range, mol wt 6500–45,000 Da (SigmaMarker^TM^, Australia) were also loaded along with the samples.

One gel was stained with Coomassie brilliant blue and the other one was stained for trypsin-inhibitor activity as described by [37] with slight modification. The principle behind this method relied on the separation of the samples into protein bands using native PAGE. The inhibitor bands on the gel upon exposure to trypsin appear as a clear band, indicating inhibition of the trypsin. Immediately after separation, the gels were rinsed in distilled water to remove excess chemicals, followed by incubation in assay buffer containing 15 mg/mL bovine trypsin for 20–30 min at room temperature. The gels were rinsed in distilled water before incubating in a staining solution for one hour without shaking. For each gel, the staining solution was prepared fresh by dissolving 0.35% *N*-acetyl-dl-phenylalanine β-naphthyl ester (APNE) in 10 mL *N*,*N*-dimethylformamide, and 0.125% tetrazoitized (zinc chloride complex) o-dianisidine (Fast blue B salt) in 100 mL of 50 mM Tris-HCl (pH 8.0) separately. These solutions were mixed before they were poured on the gel. The stained gels were rinsed in distilled water, followed by storage in 7.5% acetic acid. The presence of trypsin inhibitors was visualized as clear bands on a dark violet or pink background. The purified PI preparations were also resolved using native gel (PhastGel homogeneous 20) and denaturing gel (PhastGel gradient 8–25). All native PAGE gels were run in duplicate, stained for protein using Coomassie brilliant blue and PhastGel blue R, and their corresponding gels were treated for trypsin activity as above. The PI bands appeared as clear areas against a dark pink background. The stained gels were washed in distilled water and stored in 7.5% acetic acid before imaging. 

For nonreducing SDS-PAGE, the sample buffer (described above) was devoid of β-mercaptoethanol, but for reducing conditions, 2% β-mercaptoethanol was added. Samples for electrophoresis were incubated for 15 min at 100 °C, cooled at room temperature, clarified by centrifugation at 1000 rpm for 3 min (HermLe, Gosheim, Germany), and loaded onto the gel.

#### 4.1.3. Isoelectric Focusing (IEF) 

Isoelectric points for the canola meal crude extracts and purified samples were determined by conducting IEF on the Pharmacia Phast System (GE Healthcare Life Sciences, Uppsala, Sweden). Isoelectric focusing gels termed IEF 3–9 (which are optimized for the pH range of 3–9), were used to resolve proteins based on their isoelectric points. The reference sample used was an Amersham Biosciences broad-range p*I* calibration kit, and different proteins with well-known isoelectric points ranging from 3 to 10 were used. The protein bands were visualized using the silver staining method as per the manufacturer’s protocol (GE Healthcare Life Sciences, Uppsala, Sweden).

### 4.2. Dipeptidyl Peptidase IV (DPP-IV) Inhibitory Activity

The DPP-IV inhibitory activity of samples was measured as previously described [39] with slight modifications. All samples were prepared in a 100 mM Tris-HCl buffer. The assay was carried out in a 96-well microplate. The reaction volume of 250 μL contained 100 mM of Tris-HCl buffer (pH 8.4), 7.5 μL of DPP-IV enzyme (0.2 U/mL) and 232.5 μL of either a test sample (2 to 70 μg/mL) or the buffer as a blank. The mixture was incubated at 37 °C for 30 min, followed by the addition of 10 μL of 1.59 mM Gly-pro p-nitroanilide (substrate). The mixture was further incubated at 37 °C for 30 min. The absorbance was then measured at 410 nm in a microplate reader. The percentage inhibition of DPP-IV was calculated as:

(1) $$\mathrm{Inhibition}\;\left(\%\right)=\left(1-\frac{{\mathrm{Absorbance}}_{\mathrm{inhibitor}}\;}{{\mathrm{Absorbance}}_{\mathrm{control}}}\right)\times 100.$$

The inhibitory concentration (IC_50_) of each sample was calculated from the least squares regression line of the logarithm of the sample concentration against the DPP-IV inhibition activity.

### 4.3. Angiotensin 1-Converting Enzyme (ACE) Inhibition Activity

The ACE inhibition activity was performed in vitro as described in [40]. Prior to loading, all samples were centrifuged at 5500× *g* for 5 min to remove solid matter and incubated at 37 °C for 5 min. A 10 µL aliquot of ACE (0.25 units/mL in deionised water) and 30 µL of sample solution (0.625–10 µg/mL in 50 mM Tris–HCl, pH 7.5, containing 300 mM NaCl) were loaded into a 96-well microplate. After incubation, 150 µL of 0.88 mM FAPGG (furylacryloyl-phenylalanyl-glycyl-glycine) was added as a substrate into each well. 

The changes in absorbance at 340 nm, due to the degradation of FAPGG by ACE, and the inhibitory properties of the PIs were monitored. The ACE activity was evaluated by measuring the slope of the line representing the relationship between the absorbance at 340 nm (∆A) and five different dilutions (11 to 190 μg/mL) over 13 min. The ACE inhibitory activities (%) of the PIs were calculated as:

(2) $$\mathrm{ACE}\;\mathrm{inhibition}\;\left(\%\right)=\left(1-\frac{{\Delta A}_{\mathrm{inhibitor}}}{{\Delta A}_{\mathrm{control}}}\right)\;x\;100$$

where ∆A is the slope of the line representing the change in absorbance of a given dilution. Each ∆A value was then plotted against the respective dilution to obtain the IC_50_.

### 4.4. N-Terminal Amino Acid Sequencing

Purified samples of PIs were electrophoresed on SDS-PAGE gels under reducing conditions on a Protean II Xi Cell vertical electrophoresis system (Bio-Rad Laboratories Pty Ltd). The Coomassie brilliant blue stained visible PI bands were excised and passively eluted from the gel matrix using SDS elution buffer overnight. The samples were then loaded onto a Prosorb filter cartridge (Applied Biosystems, Carlsbad, CA, USA) and washed with 0.1% TFA (2 × 100 μL) to remove the SDS and reduce the background contamination.

The marked samples were then spotted on the polyvinylidene difluoride (PVDF) membrane and subjected to 10 cycles of Edman *N*-terminal sequencing using an Applied Biosystems 494 Procise Protein Sequencing System (Applied Biosystems, CA, USA). Performance of the sequencer was assessed routinely with 10 pmol β-Lactoglobulin standard. The resultant sequences were analyzed using the BLAST algorithm (http://www.ncbi.nlm.nih.gov, accessed on 5 November 2020) to determine the level of similarity to other proteins in the SWISS-PROT/Protein Knowledgebase (UniProtKB) database (http://www.uniprot.org, accessed on 5 November 2020).

### 4.5. Statistical Analysis

All analyses were conducted in triplicate. Data are presented as the means ± standard deviation (SD). All results were analyzed using Graph Pad Prism 5, Microsoft Excel 2016, and one-way analysis of variance (ANOVA) using SAS^®^ system for Window V8 (SAS Institute, USA). Comparisons between sample means were calculated using the Duncan Multiple Range test at a 5% probability level (*p <* 0.05).

## 5. Conclusions

This study is the first example of the identification of multiple protease inhibitor molecules from the largest commercial oilseed crop (canola). These protease inhibitors were different in two contrasting canola genotypes. Six protease inhibitor molecules in all and, of these, one from BLN-3347 and one from Rivette have not been previously reported in the literature. Several in vivo and in vitro studies have shown aqueous plant extracts derived protein hydrolysate capable of inhibiting the enzymes and transporter systems. The findings of the current investigation have shown the potential of canola PIs for the development bioactive peptides as candidates for food additives and therapeutic management of DDP-IV and ACE activities. It is therefore important that canola meal from the species *Brassica napus* should be a priority for further research funding for the application of currently available approaches, such as top-down sequencing employing proteolytic digestion and subsequent MS analysis. 

## Figures and Tables

**Table 1:** Trypsin inhibitor (TIU) activity, total protein, percent protein recovery, and purification factor at each purification step for protease inhibitors from defatted canola meal.

Steps/Characteristics	Total Protein (mg)		Specific Activity ^1^ (TIU /mg)		% Recovery ^2^		Purification Factor ^3^	
	BLN-3347	Rivette	BLN-3347	Rivette	BLN-3347	Rivette	BLN-3347	Rivette
Crude extract	4406	7560	0.013	0.008	100	100	1	1
Ammonium sulphate precipitate (AS)	1050	1498	0.44	0.06	24	20	33	7.5
Anion exchange fraction (*AEF*)	279	735	5.62	1.41	6.30	9.72	431	176
Gel filtration fraction	130	534	32	9.26	3.00	7.06	2388	1158
Second *AEF*	95	429	61	26	2.04	5.6	4633	3250

^1^ Specific activity was expressed as inhibition units per mg of protein. ^2^ Protein recovery was determined as percentage of purified inhibitor. ^3^ Purification factor was measured by specific activity values. Means showing the same letter (superscript) are not significantly different at *p* < 0.05 using Duncan’s Multiple Range Test.

**Table 2:** Identification and comparison of the amino acid sequences of protease inhibitors from two canola genotypes.

Genotype	N-Terminus	Predicted Sequence	GenBank Accession	p*I* (cal/obs)	MW (kDa) (cal/obs)
BLN-3347	APTLQGE-IK	mnipcfkfthihsvshqfcpasltqvrhasqkfrqashis trslkiaava**APTLQGE**W**IK**VEQKGGNTLSDRSSHGIAVVGDELYAFGGEFNTNDLHVFDLNTQNCTSL	XP_013643640.1 PREDICTED: epithiospecifier protein-like [*Brassica napus*]	4.49/4.00	6.4/8
	ADLVE-PKKD	maen**ADLVE**W**PKKD**KRRFLHVVYRVGDLDRTIQFYTECFGMKVLRKRDVPEEKYSNAFLGFGPETSNFVVELTYNYGVSSYDIGTGFGHFAISTQDVSKMVEAVrakggnvtrepgpvkgggsviafvkdpdgytfeliqrgptpeplcqvmlrvgdldraikfyekalgmrllrrierpeykytigmmgyaeeyesivleltynygvteytkgnayaqiaigtddvyksaevvkivnqelggkitreagplpglgtkivsfldpdgwktvlvdnedflkele	XP_013710061 PREDICTED: about 100 amino acids from determined N-terminal sequence of putative lactoylglutathione lyase [*Brassica napus*]	5.37/6.13	11.5/11
	* GQEHRIDK	No homology could be inferred	None.	--/4.90	--/18
RIVETTE	IYPSF-V	mdmatksvsslaaffilflvifempeieaqdseclkeyggdvgfgfcaprI**YPSFCV**QRCRADKGALSGKCIWGQGGNVKCLCNFCRHEPGQILSGI	XP_013688693 PREDICTED: defensin-like protein 4 [*Brassica napus*]	8.92/8.65	5.115/7
	PQGPQQRPPL	sagpfripkc rkefqqaqhl racqqwlhkq amqsgsg**PQGPQQRPPL**LQQCCNELHQEEPLCVCPTLKGASRAVKQQVRQQQGQQGQQLQQVISRIYQTATHLPKVCNIPQVSVCPFQKTMPGPS	P80208.1 **2SS3_BRANA** Napin-3 OS = *Brassica napus* PE = 1 SV = 1	9.15/9.30	9.813/15.5
	* D/AA/EE/KGKKMET/A	No homology could be inferred	None.	--/6.55	--/19

Lowercase alphabetics indicate residues upstream of the determined *N*-terminal sequence, which seemed to be chopped off as post-translational modifications. Uppercase alphabetics represent the database sequence including *N*-terminal and its downstream residues. p*I* (Isoelectric point), MW (molecular weight), cal = calculated; values were obtained on the sequence shown in upper cases using http://web.expasy.org/compute_pi/obs=observed, accessed on 5 November 2020; values obtained from Figure 5 and Figure 6. * No apparent homology that matched both the p*I* and the molecular weight could be inferred. Amino acids identical to those of previously known canola PIs are underlined. Empty areas show amino acid not determined. Amino acid abbreviations: A, alanine; P, proline; T, threonine; Q, glutamine; G, glycine; E, glutamine; C, cystine; I, isoleucine; K, phenylalanine; R, arginine; M, methionine. Source: (http://blast.ncbi.nlm.nih.gov, accessed on 5 November 2020).

## Data Availability

Data supporting reported results can be requested by contacting Mr. Bernie McInerney (bmcinern@proteome.org.au; Telephone: 02 9850 6207) at the Australian Proteome Analysis Faciity and Prof. Ian Smith (ian.smith@med.monash.edu.au; Telephone: 03 9905 1486) at Monash Proteome Facitity.

## Sample Availability

Samples of the compounds are available from the author Dr. Saira Hussain.
